# SMR peptide antagonizes mortalin promoted release of extracellular vesicles and affects mortalin protection from complement-dependent cytotoxicity in breast cancer cells and leukemia cells

**DOI:** 10.18632/oncotarget.27138

**Published:** 2019-09-10

**Authors:** Ming-Bo Huang, Jennifer Y. Wu, James Lillard, Vincent C. Bond

**Affiliations:** ^1^ Department of Microbiology, Biochemistry, and Immunology, Morehouse School of Medicine, Atlanta, GA 30310, USA; ^2^ Columbia College, Columbia University, New York, NY 10027, USA

**Keywords:** extracellular vesicles, mortalin, complement, SMR peptides, breast cancer

## Abstract

**Background:** Mortalin/GRP-75/mt-hsp70 is a mitochondrial chaperone protein, found in the cytoplasm, endoplasmic reticulum and cytoplasmic vesicles. It functions in many cellular processes such as mitochondrial biogenesis, intracellular trafficking, cell proliferation, signaling, immortalization and tumorigenesis. Thus, inhibition of mortalin is a promising avenue for cancer therapy. Previous studies in our lab have suggested that mortalin contributes to breast cancer development and progression. We showed that tumor extracellular vesicle secretion was decreased by knockdown of mortalin expression using HIV-1 Nef SMR peptides. Specifically, these peptides can block extracellular vesicle secretion and mediate cell cycle arrest in MDA-MB-231 and MCF-7 breast cancer cells.

**Aims:** This study aims to investigate further the function and mechanism of interaction of PEG-SMR-CLU and SMR-CPP peptides with the chaperone protein mortalin and to explore the effect of SMR-derived peptides and mortalin expression on extracellular vesicle release and complement dependent cell toxicity in human breast cancer and leukemia cell lines.

**Results:** Our results demonstrated additional effects reversing the tumorigenicity of these cells. First, the modified SMRwt peptides reduced the expression of the mesenchymal marker vimentin (VIM). Second, exposure to the SMRwt peptide inhibited mortalin and complement C9 expression in MDA-MB-231, MCF-7 breast cancer cells and K562 leukemia cells as measured by the Western blot analysis. Third, the SMRwt peptides blocked the cancer cells’ ability to release extracellular vesicles, which we observed blocked extracellular vesicle-mediated release of complement, re-establishing complements mediated cell death in those peptide-treated cells.

**Methods:** We developed a series of peptides derived from the Secretion Modification Region (SMR) of HIV-1 Nef protein, modified by the addition of either a cell-penetrating peptide (CPP), a positively charged arginine-rich peptide derived from HIV-1 regulatory protein Tat, or a Clusterin-binding peptide (CLU), a molecular chaperone involved in protein secretion. Both CPP and CLU peptide sequences were added at the C-terminus of the Nef SMR peptide. The CLU-containing peptides were also modified with polyethylene glycol (PEG) to enhance solubility. After treatment of cells with the peptides, we used the MTT cell viability and complement-mediated cytotoxicity assays to confirm the inhibitory role of modified SMRwt peptides on the proliferation of MDA-MB-231 and MCF-7 breast cancer cells and K562 leukemia cells. Flow cytometry was used to determine complement mediated cell apoptosis and death. Western blot analysis was used to track SMR peptides impact on expression of mortalin, vimentin and complement C9 and to measure the expression of extracellular vesicle proteins. NanoSight analysis and acetylcholinesterase (AChE) assay were used for measuring extracellular vesicles particle size and concentration and acetylcholinesterase.

**Conclusions:** Mortalin promotes cell proliferation, metastasis, angiogenesis, downregulate apoptotic signaling. Thus, mortalin is a potential therapeutic target for cancer immunotherapy. The novel SMRwt peptides antagonize the functions of mortalin, blocking tumor extracellular vesicle release and extracellular vesicle-mediated release of complement. This leads to decreases in breast cancer cell metastasis and allows standard treatment of these late stage tumor cells, thus having important clinical implications for late stage breast cancer chemotherapy. These findings support further investigation into the therapeutic value of the SMR peptide in cancer metastasis.

## INTRODUCTION

Cancer cells, including breast cancer cells and leukemia cells, release extracellular vesicles (EVs; we have previously called these exosomes, but to be exact we will hereafter refer to them as extracellular vesicles) that have been identified to contain tumor specific sets of proteins, lipids and nucleic acids. These extracellular vesicles cause immune suppression through immune cell killing or dysregulation, thereby promoting a state of immune privilege that allows for tumor growth. Therefore, extracellular vesicles play key roles in both the development of cancer and in the potential for cancer diagnosis and therapy. Cancer cells also exhibit increased resistance to complement-dependent cytotoxicity (CDC) via their ability to diminish the complement membrane attack complex (MAC) from their cell surface. The imminent participation of members of the complement cascade in several hallmarks of cancer makes it a potential target for anti-tumor treatment. Either treatment of complement-resistant K562 leukemia cells with serine protease inhibitors or the use of anti-mortalin antibodies significantly enhances K562 sensitivity to complement-mediated lysis [1, 2].

Extracellular vesicles (EV) are bioactive, small membrane vesicles (30–100 nm). They are released via fusion of multivesicular bodies (MVBs), derived from multivesicular endosomes, with the plasma membrane [3] leading to release of EVs into the extracellular medium. EVs contain microRNAs, mRNAs, DNA fragments, lipids and proteins, which can be shuttled from a donor cell to recipient cells [4]. Many different cell types including immune cells, mesenchymal cells, and cancer cells release EVs [5–8]. Several studies have demonstrated that cancer-derived EVs may contribute to the recruitment and reprogramming of constituents associated with the tumor environment of the tumor microenvironment to form a pro-tumorigenic state [9, 10]. In the tumor microenvironment, malignant cells utilize paracrine signaling initiated by adjacent stromal cells to acquire resistance to multiple types of anticancer therapies, and EVs promote cancer during metastases. We focus on the interaction of anti-tumor SMR peptides and human chaperone protein mortalin with EVs, and possible effects on K562, MDA-MB-231 and MCF-7 breast cancer cells. Further, we highlight the practical issues that should be exercised with caution to guide the development of targeting agents and therapeutic methodologies to minimize cancer resistance driven by EVs, thereby effectively controlling the early steps of disease exacerbation.

Our previous research has shown that SMR peptides could be a possible biological therapeutic for the prevention and treatment of breast cancer [8]. Breast cancer cells have been found to secrete, in a regulated manner, EVs that carry tumor antigens. Additionally, secretion of EVs by tumor cells can help to present antigens or transmit them to antigen presenting cells [11–13]. These tumor-released EVs cause immune suppression through immune cell killing or dysregulation, thereby promoting a state of immunosuppression that allows for rapid tumor growth [14, 15]. We showed that SMR peptides inhibited the growth of breast cancer cells without cytotoxic effects, and also blocked release of EVs from these cells. This effect was blocked by a knockdown of the mortalin expression [8]. This work led to the initial validation of a peptide antagonist containing a secretion domain (SMRwt-CPP), which, when taken into cells, interacts with mortalin and blocks cancer-induced EVs secretion from those cells.

The complement system plays an important role in the human immune response. Initiation of the immune response leads to assembly of terminal complement complexes followed by membrane adhesion and insertion. Subsequent complement-dependent cytotoxicity (CDC) is caused by C5b-9, the membrane attack complex (MAC), which is composed of the five complement proteins C5b, C6, C7, C8, and C9 [1, 16]. Recently, the involvement of mortalin in MAC elimination from complement resistant tumor cells has been suggested [17, 18]. Further, extracellular application of antibodies directed to mortalin re-establishes cell sensitivity to MAC-mediated lysis.

Mortalin (GRP75, mthsp70), primarily a mitochordrial protein, is also found in the endoplasmic reticulum, cytoplasm and cytoplasmic vesicles [19–21]. It is encoded by the gene HSPA9B (GeneID: 3313) localized on chromosome 5q31.1.1 and is low or undetectable in normal unstressed cells. It participates in stress response [22], mitochondrial import [23], intracellular trafficking [24] and cell proliferation [25], and is frequently upregulated in tumors [26–28]. Mortalin has been shown to contribute to the process of carcinogenesis in multiple ways including inactivation of tumor suppressor p53 protein, deregulation of apoptosis, and activation of epithelial mesenchymal transition (EMT) signaling. Mortalin/GRP75 binds to complement C9 and plays a role in resistance to complement-dependent cytotoxicity (CDC). Mortalin potentially targets the C8 and C9 complement components through its ATPase domain and inhibits C5b-9 assembly and stability [29]. Further, direct binding of mortalin to these two components (C8 and C9) of the terminal complement pathway was demonstrated with purified proteins [30]. Mortalin also plays a role in cell resistance to CDC and in MAC elimination [2, 19]. Complement-dependent cytotoxicity is an essential immune effector mechanism employed to eliminate pathogens and abnormal cells from the body [30, 31]. CDC is activated in cancer patients upon injection of therapeutic anticancer Abs and participates in the overall fight against the cancerous cells [32]. Complement-resistant K562 cells are completely resistant to lysis by homologous human serum, unless treated first with sensitizing anti-serum [33]. Treatment of complement-resistant K562 cells with serine protease inhibitors enhances their sensitivity to complement-mediated lysis. K562 cells release EVs with similar characteristics to reticulocyte EVs and are therefore a useful model to study the secretion of these small vesicles from erythroid cells [34].

In the current investigation, we focused on mortalin a mitochondrial chaperone and a member of the heat shock protein (Hsp) 70 family, which is enriched in human cancer cells, and plays a significant role in maintaining mitochondrial function [35]. Mortalin overexpression has been detected in various tumor types [36]. It plays a role in carcinogenesis and confers chemotherapeutic drug resistance [27, 37–39]. Several studies have shown that mortalin has multiple important functions contributing to continued proliferation of cancer cells, including mitochondrial biogenesis, ATP production, anti-apoptosis, chaperoning, inactivation of tumor suppressor p53 and p13K/AKT activities [40–42]. Suppression of the gene HSPA9 expression or interference with interaction would be a therapeutic strategy against cancer. Indeed, knockdown of the gene HSPA9 by ribozyme or small interference RNA (siRNA), including treat with MKT-077 reduces cell growth and viability of human cancer cells [8, 40, 43–47]. MKT-077, a cationic rhodacyanine dye analogue [48], binds to mortalin and dissociates it from p53, thus restoring p53 transcriptional activity and apoptosis. Cancer cells, which have a higher mitochondrial membrane potential than normal cells, show higher sensitivity to MKT-077 [49–52]. The purpose of this study was to use breast cancer cells and K562 cells to study the effect of the SMR peptide on CDC and MAC. To block mortalin synthesis, MDA-MB-231, MCF-7 breast cancer cells and K562 leukemia cells were transfected with mortalin-specific siRNA (HSPA9). To inhibit mortalin activity, MDA-MB-231, MCF-7 and K562 cells were treated with SMR peptide and MKT-077. Our results show that both treatments reduce the EV process and sensitize MDA-MB-231, MCF-7 and K562 cells to complement-dependent cytotoxicity. The data supports a role of mortalin in protection of MDA-MB-231, MCF-7 and K562 cells from complement, possibly by interacting with complement C9 and affecting MAC formation and stability.

## RESULTS

### SMRwt peptide inhibited cell proliferation and growth of MCF-7, MDA-MB-231 breast cancer cells and K562 leukemia cells

**Figure 1:**
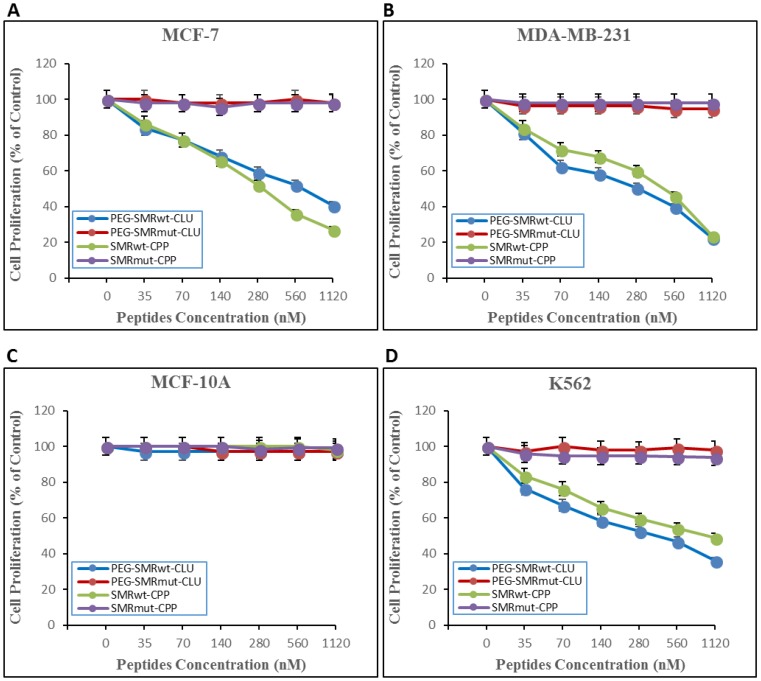
SMRwt-CPP peptides affect proliferation of MCF-7 and MDA-MB-231 breast cancer cells and K562 leukemia cells, but not non-tumorigenic MCF-10A cells. Cells were incubated with peptides at varying dosages (0–1120 nM) for 24 hours at 37^°^ C, after which proliferation was measured by MTT assay. Results of three independent experiments are shown. (**A**) proliferation of MCF-7 breast cancer cells. PEG-SMRwt-Clu (blue dots) IC50 was 600 nM/mL and SMRwt-CPP (green dots) IC50 was 300 nM. Red dots indicate PEG-SMRmut-CLU peptide and purple dots indicate SMRmut-CPP peptide as control. (**B**) proliferation of MDA-MB-231 breast cancer cells. PEG-SMRwt-CLU (blue dots) IC50 was 280 nM and SMRwt-CPP (green dots) IC50 was 540 nM. (**C**) proliferation of non-tumorigenic MCF-10A cells as control cells. (**D**) proliferation of K562 leukemia cells. PEG-SMRwt-CLU (blue dots) IC50 was 560 nM and SMRwt-CPP (green dots) IC50 was 1120 nM. Red dots indicate PEG-SMRmut-CLU peptide and purple dots indicate SMRmut-CPP peptide as control.

Effects of different dosages of the PEG-SMRwt-Clu peptide and SMRwt-CPP peptide on MCF-7, MDA-MB-231, and K562 were quantified via MTT assay. MCF-1, MDA-MB-231 and K562 cultures were treated for 24 hours with increasing concentrations (35 nM, 70 nM, 140 nM, 280 nM, 560 nM and 1120 nM) of either SMRwt-CPP peptide or the negative control peptide SMRmut-CPP. Growth was inhibited in all cell’s tests in a dose-dependent manner by SMRwt-CPP peptide, but not the negative control peptide. The IC50 dosage was estimated from fitted response curves. Each curve describes how the percentage of surviving cells depends on the dose level, with the IC50 giving 50% inhibition. The inhibition observed was dose dependent, with a typical sigmoidal shape of dose response, corroborating validity of the observations. The IC50 of the PEG-SMRwt-Clu peptide against MCF-7 cells was 600 nM and SMRwt-CPP against MCF-7 was 300 nM (Figure 1A), PEG-SMRwt-Clu peptides against MDA-MB-231 was 540 nM and SMRwt-CPP peptide against MDA-MB-231 was 280 nM (Figure 1B), and PEG-SMRwt-Clu peptide against K562 was 560 nM and SMRwt-CPP peptides was 1120 nM (Figure 1D). Non-tumorigenic MCF-10A cells were examined as the cell control and were not affected by PEG-SMRwt-Clu and SMRwt-CPP peptides (Figure 1C).

### SMRwt peptides, MKT-077, omeprazole and DMA blocked extracellular vesicle release in MCF-7, MDA-MB-231 breast cancer cells, and K562 leukemia cells

**Figure 2:**
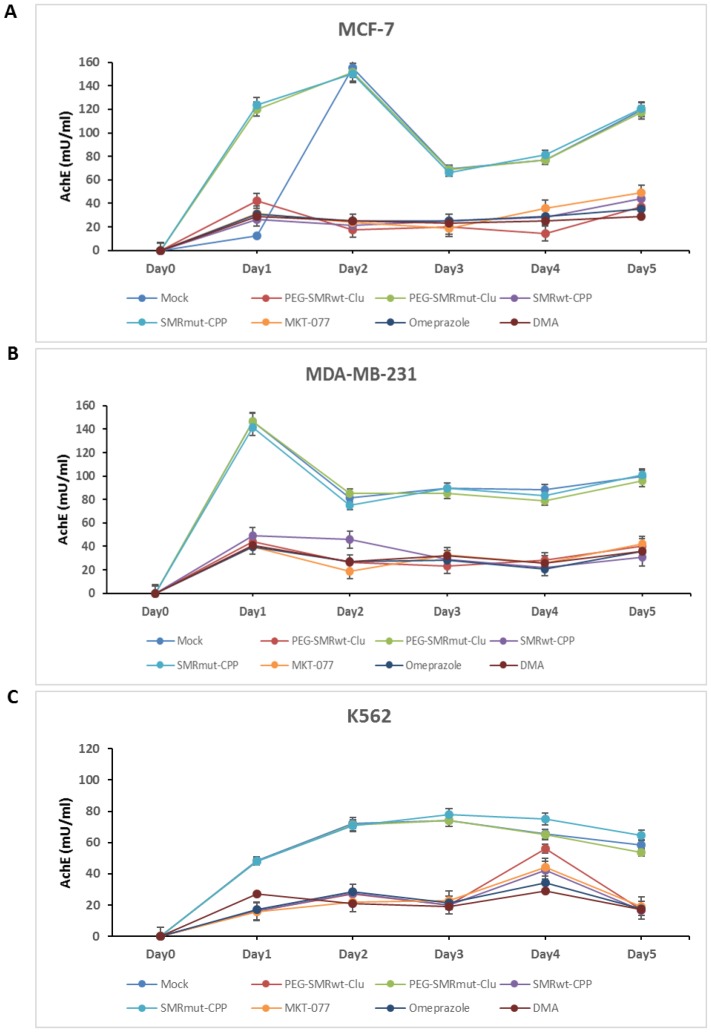
PEG-SMRwt-Clu peptide, SMRwt-CPP peptide, MKT-077, omeprazole and DMA antagonist reduced extracellular vesicle (EV) release from MCF-7, MDA-MB-231 breast cancer cells and K562 leukemia cells. Culture cells were treated with IC50 PEG-SMRwt-Clu peptide, PEG-SMRmut-Clu peptide, SMRwt-CPP peptide, SMRmut-CPP peptide, 1 μM MKT-077, 200 μM omeprazole and 300 μM DMA for five days at 37^°^ C. Level of EV’s released from (**A**) MCF-7 cells, (**B**) MDA-MB-231 cells, and (**C**) K562 cells, respectively, determined by acetylcholinesterase (AchE) assay.

It has already been published by our group that PEG-SMRwt-Clu peptide blocks EV release in breast cancer cells [8]. In this study, we examined effects of PEG-SMRwt-Clu peptide and SMRwt-CPP peptide, and the mortalin inhibitors MKT-077, omeprazole and DMA. We performed acetylcholinesterase (AchE) assays, NanoSight analysis and Western blot analysis to characterize EVs released from MCF-7 and MDA-MB-231 human breast cancer cells and K562 leukemia cells treated for five days at 37° C with the IC50 for various peptides, or 1 μM MKT-077, 200 μM omeprazole and 300 μM DMA. On comparing the number of EVs released from these different human cancer cell lines, we observed that PEG-SMRwt-Clu peptide, SMRwt-CPP peptide, MKT-077, omeprazole and DMA mortalin inhibitors all reduced the number of EVs released, and this was maintained during the five days of treatment. Figure 2A shows the results obtained when MCF-7 breast cancer cells were treated with the 5 different molecules and 3 controls. When AChE activity in EVs was assayed and the results compared, we observed a rise in the concentration of AChE in days 1 and 2 in MCF-7 cells exposed to the controls (Mock, PEG-SMRmut-Clu peptide and SMRmut-CPP peptide); the concentrations at day 2 was 155.13 mU, 151.5 mU and 150.06 mU respectively. At day 3 we observed a rapid decrease in AChE concentrations (69.15 mU, 69.0 mU and 66.05 mU), while observing increases in AChE concentration on day 4 and day 5. For the experimental molecules, AChE concentrations were maintained at low levels throughout day 1 to day 5 and maintained lower concentrations (average level: 31 mU, 22 mU/mL, 22 mU 26 mU and 26 mU). Figure 2B displays the results obtained for treated MDA-MB-231 cells for AChE activity in EVs**.** An increase in the AChE concentration was observed for the control groups in day 1 was 146.52 mU, 146.79 mU and 141.47 mU, respectively, followed by decreases in AChE concentrations in days 2-5. Alternatively, in the experimental treatment groups, AChE levels were maintained at low concentrations throughout day 1 to 5 (average level: 42 mU, 29 mU, 29 mU, 25 mU and 37 mU). Figure 2C displays results obtained for K562 leukemia cells treated as were the other two tumor cells. For the control groups, a rise in AChE concentration was observed across the 5 days, with day 1 to day 5 showing concentrations of average level were 48 mU, 71 mU, 75 mU, 69 mU and 59 mU, respectively. Again, in the experimental treatment groups, AChE concentrations were maintained at low levels across the 5 days treatment with days 1-3, and 5 being 18 mU, 25 mU, 21 mU and 18 mU with day 4 showing a large increase, 41 mU. These results indicated that EV release was inhibited by all experimental treatments (Figure 2).

**Figure 3:**
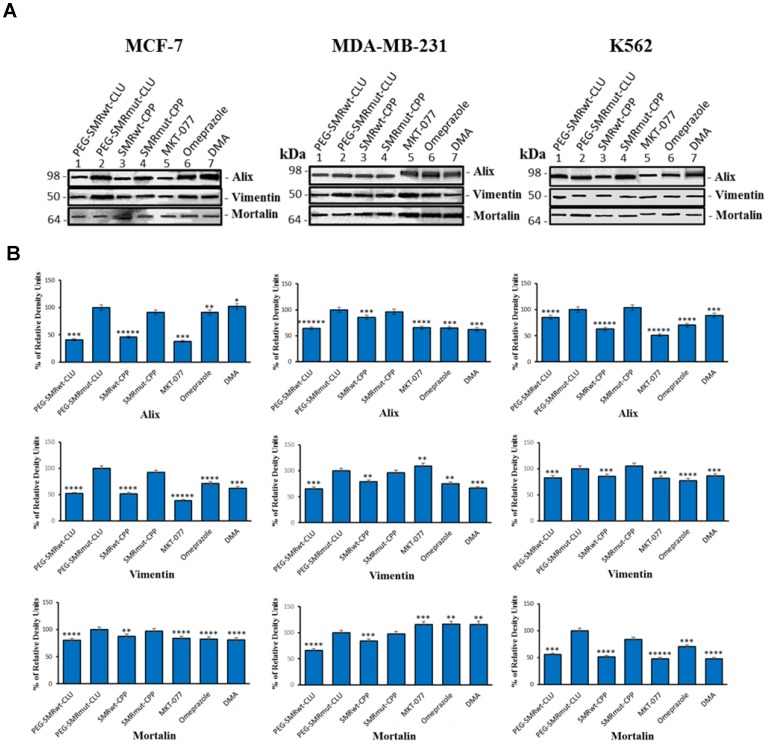
Mortalin, vimentin and EV–specific proteins can be detected in extracellular vesicles of MCF-7 breast cancer cells. Culture cells were treated with IC50 PEG-SMRwt-Clu and SMRwt-CPP peptides, 0.25 μM MKT-077, 200 μM omeprazole and 300 μM DMA at 37^°^ C for 48 hours. Western blot analysis using anti-mortalin (Grp-75) antibody, anti-vimentin antibody and anti-Alix antibody to evaluate mortalin, vimentin and EV’s expression in (**A**) gel images of EV’s from MCF-7, MDA-MB-231 breast cancer cells and K562 leukemia cells, (**B**) bands intensity of EV’s from MCF-7, MDA-MB-231 breast cancer cells and K562 leukemia cells. Data represent the mean ± SD of three independent experiments. Significant differences relative to treatment with peptides are indicated as follows: ^*^ *p* < 0.01, ^**^ *p* < 0.001, ^***^ *p* < 0.0001, ^****^ *p* < 0.00001, ^*****^ *p* < 0.000001. Densitometry analysis relative intensity of bands. Data represent the quantitative results from Western blots.

### SMRwt peptides and inhibitors modulated EV protein content in MCF-7, MDA-MB-231 and K562 cells

Western blot analysis was used to detect targeted EV proteins in control treated (SMRmut), and experimental molecule treated (SMRwt peptides, MKT-077, omeprazole, DMA) cultures (MCF-7, MDA-MB-231, and K562 cells). The proteins Alix, mortalin and vimentin were found in EVs isolated from all treated cell lines screened. In MCF-7 cells, Alix expression in EVs was significantly decreased in PEG-SMRwt-Clu, SMRwt-CPP and MKT-077 treatments, while the same cell treated by omeprazole and DMA displayed no significant changes, looking like the negative control, SMRmut peptide. Vimentin expression in EVs was significantly decreased due to all treatments (PEG-SMRwt-Clu, SMRwt-CPP, MKT-077, omeprazole and DMA) as compared to the negative controls. Alternatively, mortalin expression in EVs was with a slight decrease in the PEG-SMRwt-Clu peptide, SMRwt-CPP peptide, MKT-077, omeprazole and DMA treatment. For MDA-MB-231 cells, Alix expression in EVs was observed to be significantly decreased because of PEG-SMRwt-Clu and SMRwt-CPP treatment and other three inhibitors. Vimentin expression in EVs was significantly decreased in PEG-SMRwt-Clu, SMRwt-CPP, omeprazole and DMA treatments, while no significant change was observed in MKT-077 treatments. Mortalin expression was significantly decreased because of PEG-SMRwt-Clu and SMRwt-CPP treatment, with no change observed for the three inhibitors treatment. Finally, in K562 cells, Alix expression in EVs was significantly decreased due to all experimental treatments (PEG-SMRwt-Clu, SMRwt-CPP, MKT-077, omeprazole and DMA). Vimentin expression in EVs was significantly decreased due to PEG-SMRwt-Clu peptide, SMRwt-CPP peptide, MKT-077, omeprazole and DMA treatment. Mortalin expression was significantly decreased due to all experimental treatments (PEG-SMRwt-Clu peptide, SMRwt-CPP peptide, MKT-077, omeprazole and DMA). This set of data suggests that EV numbers and EV composition are modulated differently depending on the treatment molecule (Figure 3).

### MKT-077, DMA and omeprazole-targeted mortalin interactions reactivated apoptotic pathway and affected paclitaxel and cisplatin apoptosis in cancer cells

**Figure 4:**
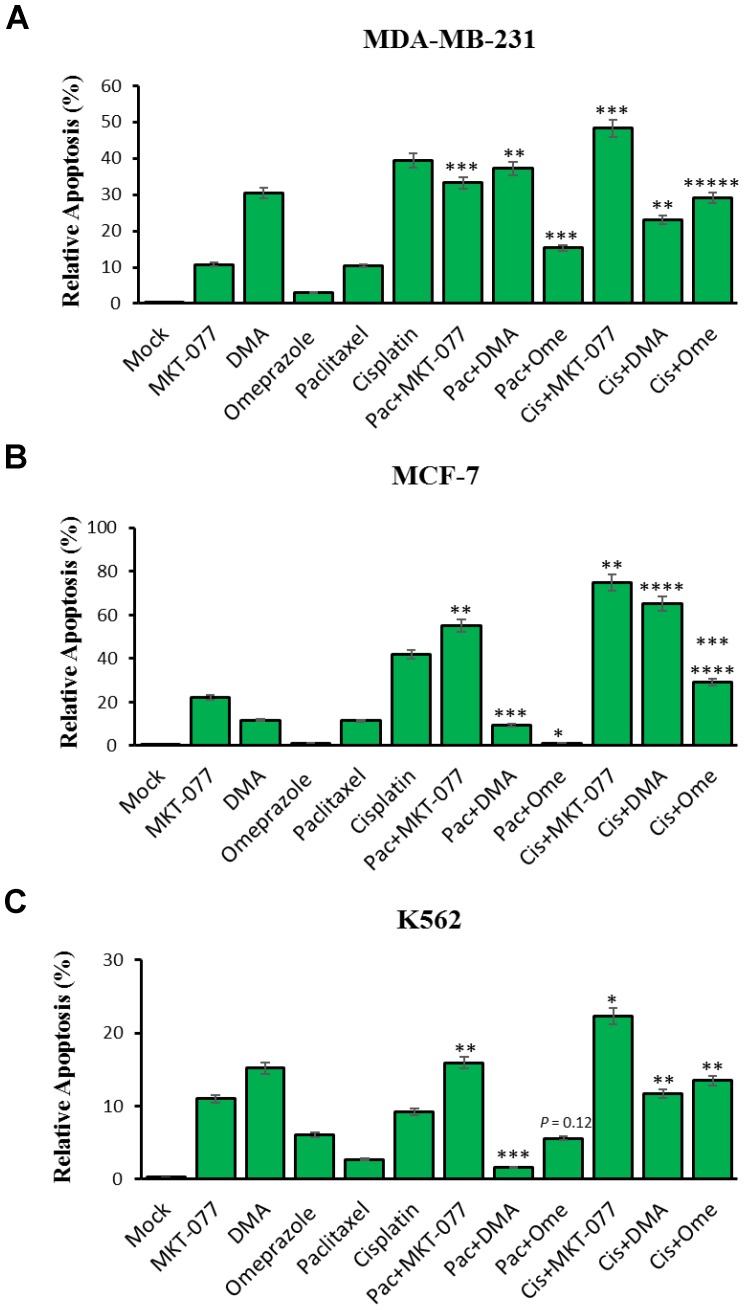
MKT-077, Dimethylallyl (DMA) and omeprazole-targeted mortalin effects on paclitaxel and cisplatin-induced apoptosis in cancer cells. Cells were treated for 48 hours with 0.25 μM MKT-077, 300 μM DMA and 200 μM omeprazole alone or combined with 1.6 μM paclitaxel or 2 mg/mL of cisplatin. (**A**) Relative level of apoptosis from MDA-MB-231 cells, (**B**) relative level of apoptosis from MCF-7 cells, and (**C**) relative level of apoptosis from K562 leukemia cells, as determined by flow cytometry. Error bars represent mean ± SD of four independent experiments. Significant differences relative to inhibitors are indicated as follows: significant differences relative to MKT-077 versus MKT-077 combined paclitaxel and cisplatin, DMA versus DMA combined paclitaxel and cisplatin, and omeprazole versus omeprazole combined with paclitaxel and cisplatin, respectively, are indicated as follows: ^**^ *p* < 0.005, ^***^ *p* < 0.0005 and ^*****^ *p* < 0.000005 for MDA-MB-231 cells; ^*^ *p* < 0.05, ^**^ *p* < 0.005, ^***^ *p* < 0.0005, ^****^ *p* < 0.00005 and ^*******^ *p* < 7.75E-08 for MCF-7 cells; ^*^ *p* < 0.05, ^**^ *p* < 0.005, ^***^ *p* < 0.0005 and *p* = 0.12 for K562.

We examined the effect of mortalin inhibitor drugs (MKT-077, DMA, and omeprazole) alone and in combination with paclitaxel or cisplatin to promote apoptosis in the three tumor cell lines MBA-MD-231, MCF-7, and K562. Cells were treated as follows: 0.25 μM MKT-077, 300 μM DMA and 200 μM omeprazole alone or combined with 1.6 μM paclitaxel or 2 mg/mL of cisplatin for 48 hours and assayed for apoptosis by flow cytometry (Figure 4). The results showed (*i*) increased apoptosis relative to the MKT-077 alone (10.7%) versus incubation with paclitaxel (33.25%) and cisplatin (48.25%) on MDA-MB-231 cells, increased apoptosis relative to the MKT-077 alone (22.03%) versus with paclitaxel (55.18%) and cisplatin (75.03%) on MCF-7 cells and increased apoptosis relative to the MKT-077 alone (11.0%) versus with paclitaxel (15.9%) and cisplatin (22.33%) on K562 cells. However, no significant changes were observed with paclitaxel on MCF-7 cells and K562 cells. (*ii*) Increased apoptosis with DMA alone (30.43%) versus incubated with paclitaxel (37.18%) but decreased with cisplatin (23.03%) on MDA-MB-231 cells; increased apoptosis relative to DMA alone (11.83%) versus with cisplatin (65.3%), but decreased apoptosis with paclitaxel (9.43%) on MCF-7 cells; and decreased apoptosis relative to the DMA alone (15.2%) versus with paclitaxel (1.63%) and cisplatin (11.7%) on K562 cells. No significant changes were observed with cisplatin in MDA-MB-231 cells, with paclitaxel on MCF-7 cells, and with paclitaxel and cisplatin in K562 cells. (*iii*) Increased apoptosis was observed for omeprazole alone (3.0%) versus incubated with paclitaxel (15.33%) and cisplatin (29.05%) on MDA-MB-231 cells; increased apoptosis for omeprazole alone (1.1%) versus combined with cisplatin (29.05%) on MCF-7 cells; and increased apoptosis relative to the omeprazole alone (6.1%) versus combined with cisplatin (13.5%) on K562 cells. No significant changes were observed with paclitaxel on MCF-7 cells and K562 cells although we observed a very small, synergistic effect of omeprazole in combination with either drug.

### SMRwt peptides blocked both mortalin-driven extracellular vesicle release and complement-dependent cytotoxicity

**Figure 5:**
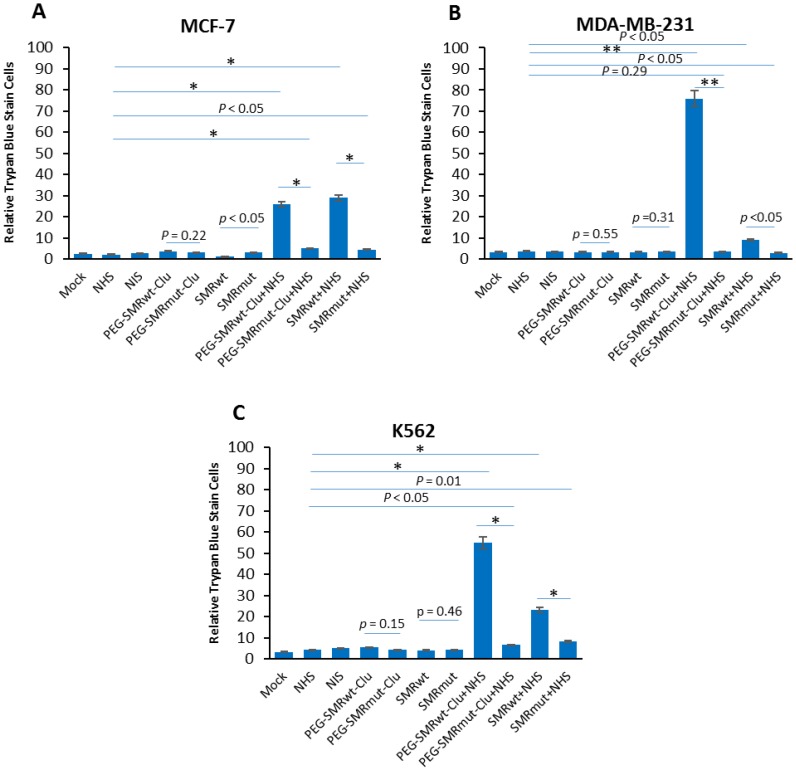
SMRwt peptides blocked mortalin-driven EV release of mortalin and induced complement-mediated cytotoxicity in K562, MCF-7 and MDA-MB-231 cultures. Cells were treated with 600 nM PEG-SMRwt-Clu peptide or 300 nM SMRwt-CPP peptide alone in MCF-7, 280 nM PEG-SMRwt-Clu or 540 nM SMRwt-CPP alone in MDA-MB-231 and 560 nM PEG-SMRwt-Clu or 1120 nM SMRwt-CPP alone in K562 for 60 min at 37^°^ C, followed by treatment with 1 μg/mL anti-CXCR4 antibody and 50 μL NHS or HIS for additional 60 min at 37^°^ C. PEG-SMRmut-Clu peptide and SMRmut-CPP peptide as control. Percent stained cells shown is representative of 3 independent experiments. These results show that PEG-SMRwt-Clu peptide and SMRwt-CPP peptide present NHS, via block mortalin-driven EV pumping out of complement and induced complement-mediated cytotoxicity, but not SMR peptides alone. (**A**) MCF-7 breast cancer cells, (**B**) MDA-MB-231 breast cancer cells, and (**C**) K562 leukemia cells, as determined by trypan blue. Error bars represent mean ± SD of four independent experiments. Significant differences relative to PEG-SMRwt-Clu peptide and SMRwt-CPP peptide are indicated as follows: ^*^ *p* < 0.01, ^**^ *p* < 0.001.

Complement-mediated cytotoxicity is a normal cellular mechanism for ridding the host of these compromised tumor cells. Mortalin/GRP75 has been shown to bind complement factor C9 and play a major role in development of resistance to complement-dependent cytotoxicity via mortalin induced exocytosis of the MAC via EVs [2]. We predict that SMR peptide driven mortalin sequestration and subsequent disruption of its functions will increase cell sensitivity to complement-induced cell death. To examine this, we treated MCF-7, MDA-MB-231, and K562 cells with PEG-SMRwt-Clu and SMRwt-CPP peptides and then performed complement-mediated cell toxicity assays. Cells were treated with anti-CXCR4 antibody for 30 min at 4° C and then with normal human serum (NHS), or heat inactivated normal human serum (HIS) [16], plus or minus SMR peptides for 60 min at 37° C. In the presence of NHS, both of SMRwt peptides significantly induced tumor cell death via complement. In MCF-7 cells (Figure 5A), cell death was observed going from 2.1% (bar 4) to 26% (bar 8) in cultures treated with PEG-SMRwt-Clu minus or plus NHS, and 2.1% (bar 6) to 29% (bar 10) in cultures treated with SMRwt-CPP minus or plus NHS. In MDA-MB-231 cells (Figure 5B), cell death was observed going from 3.6% (bar 4) to 76% (bar 8) in cultures treated with PEG-SMRwt-Clu minus or plus NHS, and 3.6% (bar 6) to 9% (bar 10) in cultures treated with SMRwt-CPP minus or plus NHS. In K562 cells (Figure 5C), cell death was observed at 4.3% (bar 4) to 55% (bar 8) in cultures treated with PEG-SMRwt-Clu minus or plus NHS, and 4.3% (bar 6) to 23% (bar10) in cultures treated with SMRwt-CPP minus or plus NHS. Thus, SMRwt peptide-induced mortalin sequestration and function disruption is linked to complement-mediated sensitivity and cell death. These data show that peptide blocking of the tumor cell’s ability to secrete complement components restores complement mediated cell toxicity and cell killing via the complement mediated mechanism.

### Treatment with SMR peptides reduced expression of mortalin and C9 in extracellular vesicles released from MCF-7, MDA-MB-231, and K562 cells

**Figure 6:**
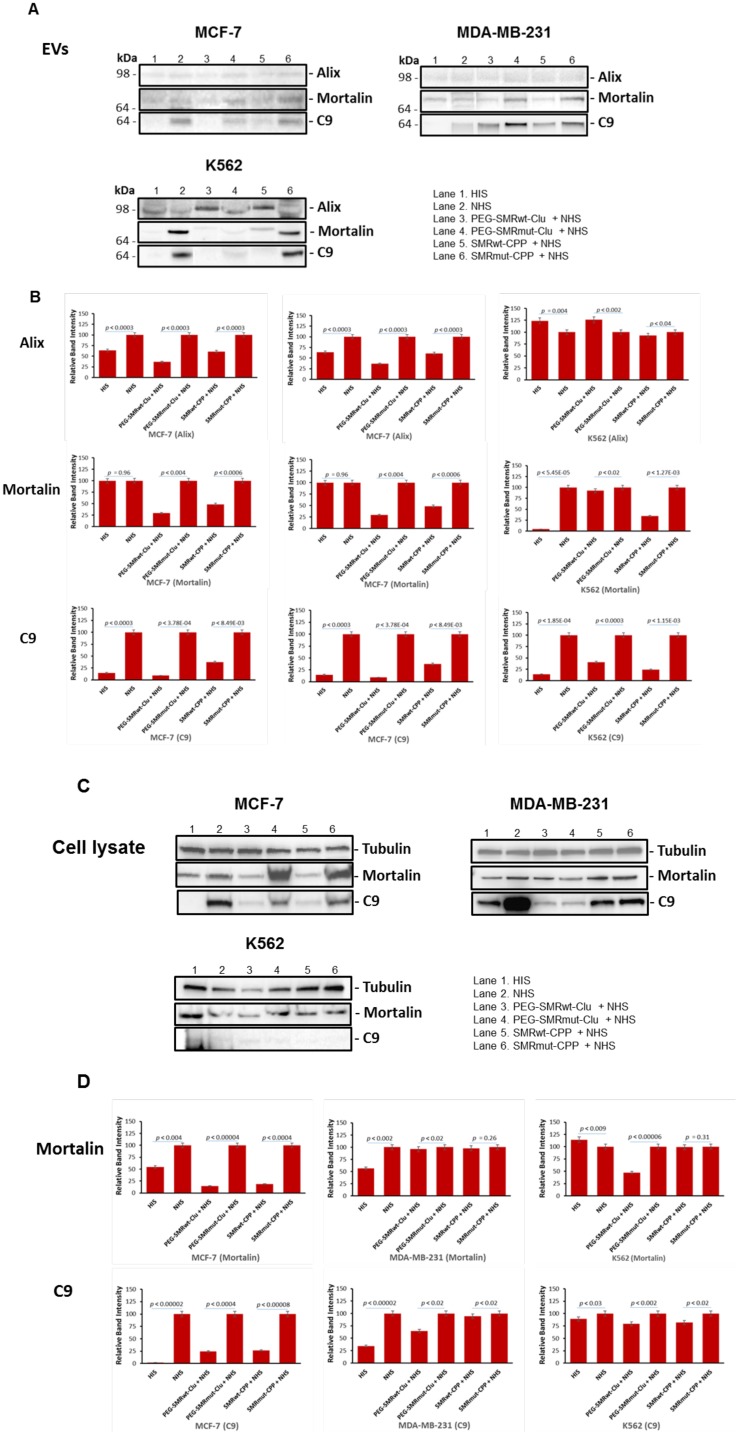
Levels of mortalin and C9 in extracellular vesicles and cell lysates from MCF-7, MDA-MB-231 and K562 cells were reduced after treatment with SMR peptides. Cells were treated with 600 nM PEG-SMRwt-Clu peptide or 300 nM SMRwt-CPP peptide alone in MCF-7 cells and 280 nM PEG-SMRwt-Clu peptide or 540 nM SMRwt-CPP peptide alone in MDA-MB-231 cells for 60 min at 37^°^ C, followed by treatment with 1 μg/mL anti-CXCR4 antibody and 50 μL NHS or HIS for 60 min at 37^°^ C, washing with PBS and the addition of fresh Exo-free medium incubated at 37^°^ C for 60 min. Supernatants were collected and EV’s were isolated. Released EVs were isolated and analyzed by SDS-PAGE and western blotting with anti-Alix, anti-Mortalin and anti-C9 antibodies. (**A**) Western blot images of EVs; (**B**) Western blot analysis of the Western images shown in (**A**); (**C**) Western blot images of comparable cell lysates; (**D**) Western blot analysis of the Western images in (**C**); Band intensities were compared to NHS controls. Results are representative of three independent experiments.

MCF-7, MDA-MB-231 breast cancer and K562 leukemia cells were treated with SMRwt peptides for 60 min at 37° C, then treated with anti-CXCR4 antibody (to induce the complement cascade) in NHS or HIS for 60 min at 37° C. Culture supernatants were clarified at 2000 × g for 10 min to remove cell debris, and then EVs were isolated. The extracellular vesicles pellets were subjected to SDS-PAGE and Western blotting and analysis was performed with anti-mortalin antibodies, anti-Alix antibodies and anti-C9 antibodies. The cell pellets were collected, subjected to SDS-PAGE and Western blotting, and analysis with the same antibodies used in analysis of the EVs. The results, shown in Figure 6, indicated the presence of Alix, mortalin and C9 in extracellular vesicles released from MCF-7, MDA-MB-231 and K562 cells treated with SMRwt peptides. The data showed that after treatment with PEG-SMRwt-Clu peptide and NHS, the mortalin levels in extracellular vesicles compared to the NHS-treated controls were 29.42% in MCF-7 and 46.24% in MDA-MB-231cells, with little change in K562 cells. Treatment with SMRwt-CPP peptide and NHS resulted in reduction of mortalin expression levels to 48.43% in MCF-7, 27.52% in MDA-MB-231 and 34.42% in K562. For the PEG-SMRwt-Clu peptide and NHS, the C9 levels in extracellular vesicles were reduced to 9.04% in MCF-7, 59.19% in MDA-MB-231, and 40.25% in K562. Treatment with SMR-CPP peptide and NHS resulted in C9 levels of 37.46% in MCF-7, 56.27% for MDA-MB-231 and 24.33% for K562 extracellular vesicles. This provides more evidence that the SMR-mortalin interaction modulates extracellular vesicle content. Further, levels of the extracellular vesicle marker, Alix, show that, while treatment with SMR peptides inhibited release of extracellular vesicles from cells, this did not entirely account for the decrease in measured levels of mortalin and C9, suggesting that packaging of these proteins into EVs was altered by SMR peptide treatment (Figure 6A and 6B). Mortalin, and C9 protein were all decreased by both SMRwt peptides versus SMRmut peptide in MCF-7 cells and the pattern was similar in both cell lysates and in the released EVs. In 231 and K562 cells, C9 shows a similar albeit less significant pattern when comparing effect of SMRwt peptides versus SMRmut peptide. Mortalin in those two cell lines does not show a distinctive pattern of change. Despite the changes in C9 and Mortalin, tubulin controls are essentially unaltered by the SMR peptides suggesting that cellular protein synthesis is not compromised (Figure 6C and 6D).

### Transient transfection with mortalin small interfering RNA (siRNA) reduced mortalin level and extracellular vesicle secretion, and induced complement-mediated cytotoxicity in K562, MCF-7 and MDA-MB-231 cells

**Figure 7:**
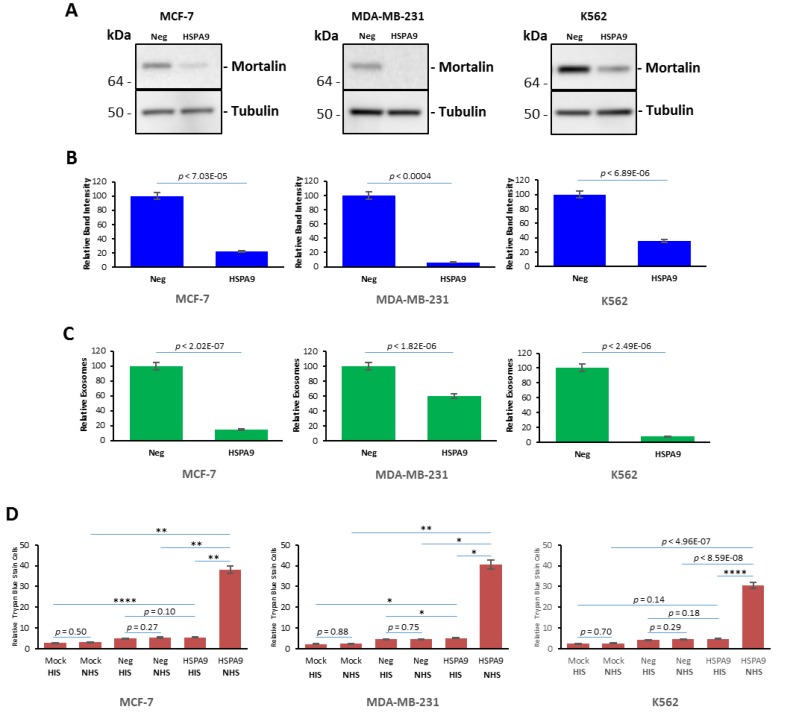
Knockdown of mortalin expression induced complement-mediated cytotoxicity in K562, MCF-7 and MDA-MB-231 cultures. Cells were transfected with double-stranded siRNAs using Amaxa’s Nucleofector kit (Lonza Walkersville Inc., Walkersville, MD), according to the manufacturer’s protocol. Transfection of plasmids was done with Amaxa Biosystems Nucleofector II as recommended. The small interfering RNA for HSPA9 was identical to that previously described (Huang *et al*, 2017). The kit also contained a corresponding pcDNA6.2-GW/miR-neg control plasmid (miR-neg) predicted not to target any known vertebrate gene, which was used as a negative control. Following transfection, the cells were incubated with culture medium at 37° C for 72 hours. (**A**) After 72 hours, the cell lysates were prepared and analyzed by SDS-PAGE and Western blotting with anti-mortalin and anti-tubulin antibodies. (**B**) Relative to band intensity, (**C**) Relative numbers of EVs released by MCF-7, MDA-MB-231 and K562 cells respectively, as determined by NanoSight measurement. (**D**) MDA-MB-231 cells, MCF-7 cells and K562 cells transfected with siRNA for 72 hours, and then treated with anti-CXCR4 antibody at 4° C for 30min, and treated with HIS or NHS at 37^°^ C for 60 min. After 60 min, cell lysis was determined by trypan blue. Error bars represent mean ± SD of four independent experiments. Significant differences relative to siRNA-Neg transfected cells with NHS and mortalin siRNA transfected cells treated with NHS are indicated as follows: ^*^ *p* < 0.01, ^**^ *p* < 0.001.

To further show the significance of mortalin in the protection of the tumor cells from complement, we looked to affect mortalin levels through a non-peptide inhibitor mechanism, siRNA knockdown. After 24 hours in EV-free media at 37° C, cells were transfected with double-stranded siRNAs [7, 8], with other cells treated without siRNA as a negative control. The siRNA-transfected cell cultures were incubated for 72 hours at 37° C. Mortalin protein was measured via western blotting with anti-mortalin antibody, obtaining protein bands with relative intensities compared to control cells as shown in Figure 7A and 7B; 21.86%/MCF-7, 6.28%/MDA-MB-231, and 35.3%/K562. EVs were isolated from the cell supernatants and analyzed for concentration and size distribution via NanoSight LM10 Nanoparticle Tracking Analysis (NTA). With NTA, particles are automatically tracked and sized, based on Brownian motion and the associated diffusion coefficient. Before analysis of the samples by NTA, we made sure that salt aggregates from PBS did not contribute to background and that equipment was free of contaminant particles. In MCF-7 cultures the siRNA-Neg controls contained 3.91 × 10^9^ particles/mL, and the siRNA mortalin culture contained 5.79 × 10^8^ particles/mL (*p* < 2.02E-07). In MDA-MB-231 cultures the siRNA-Neg controls contained 5.71 × 10^9^ particles/ml, while the siRNA mortalin culture contained 3.46 × 10^9^ particles/mL (*p* < 1.82E-06). In K562 cells, the siRNA-Neg controls contained 3.44 × 10^9^ particles/mL, and siRNA mortalin culture had 2.58 × 10^8^ particles/mL (*p* < 2.49E-06). The EV numbers from all cultures transfected with mortalin siRNA EVs were lower than the linked negative control/siRNA-Neg transfected cultures. For all cultures, NTA estimated the size of the vesicles to be in the range of 30 to 47 nm (Figure 7C). As shown in Figure 7A–7C, mortalin siRNA significantly reduced mortalin protein levels in the three cell lines, with western blotting and NanoSight analysis giving similar results. Finally, siRNA-transfected were then treated with anti-CXCR4 antibody for 30 minutes at 4° C followed by incubation with either NHS or NIS for 60 minutes at 37° C in order to see if there was a change in the level of complement-mediated toxicity. The data for this experiment, shown in Figure 7D, indicated that siRNA mortalin silencing increased complement mediated cytotoxicity in these cultures as shown by dramatic increases in cell death for the HSPA9/NHS bar on each graph (38.02%/MCF-7, 40.55%/MDA-MB-231 and 30.54%/K562).

**Figure 8:**
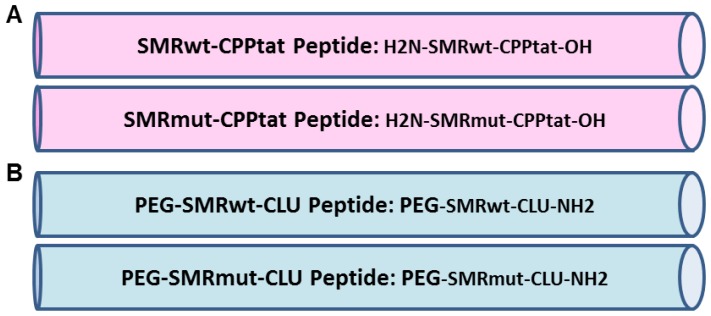
Development of SMR peptides antagonist for cancer cells. Schematic representation of the wild-type antagonist (SMRwt) and negative control (SMRmut) synthetic peptides derived from HIV-1 Nef Secretion Modification Region (SMR). Our previous extensive genetic analysis of Nef revealed several highly conserved N-terminal domains that were necessary and sufficient for Nef-induced exNef secretion. One of these domains, the novel SMR (^66^VGFPV^70^), is highly conserved across all HIV-1 clades, HIV-2, and SIV. (**A**) SMR peptides contain cell-penetrating peptides derived from the HIV-1 Tat protein (CPP-Tat) at the C-terminus; and (**B**) SMR peptides contain a poly-ethylene-glycol (PEG) at N-terminus and a clusterin (Clu) at C-terminus.

## DISCUSSION

In this study we investigated the interaction of HIV-1 Nef SMRwt peptides with mortalin in breast cancer cells. Treatment of tumor cells with SMRwt peptides decreased the release of extracellular vesicles (EVs) from tumor cells and re-sensitized the cells to complement-mediated lysis.

Specifically, the SMRwt peptides antagonized cell proliferation and reduced growth of MCF-7, MDA-MB-231 breast cancer cells and K562 leukemia cells. EV release was antagonized by all experimental treatments. The SMR peptides worked comparably to the three chemical compound inhibitors. Non-tumorigenic MCF-10A cells were examined as the cell control and were not affected by PEG-SMRwt-Clu and SMRwt-CPP peptides.

Treatment with the SMR peptide antagonists reduced the concentration of mortalin and C9 in EVs released from the two breast tumor lines and the lymphocytic tumor line. Both the SMRwt peptides and chemical inhibitors also were found to modulate the EV protein content in MCF-7, MDA-MB-231 and K562 cells with the effects being variable by cell type. We expected to see increased amounts of the two proteins mortalin and C9 in the comparable cell lysates of peptide treated cultures. Interestingly, we observed the opposite effect, with the comparable cell lysates displaying a similar pattern of SMRwt peptide-reduced expression of mortalin and C9. Alternatively, the tubulin controls displayed unaltered concentrations upon treatment with the same peptides. This evidence suggests, first, that the peptides had no effect on overall cellular protein synthesis. Secondly, a likely explanation of the reduced concentrations of C9 and mortalin in the cell lysates is that the SMR peptides affect (reduce) packaging of specific proteins into the released EV population. For example, the peptides could be redirecting those same proteins to be degraded in the lysosomes. These initial observations will be followed up on by examining other EV and non-EV proteins to test whether similar changes take place. Further, we can directly measure the routing of C9 to the lysosomes with or without peptide treatment.

It had already been shown that DMA and omeprazole blocked tumor-driven EV release of the complement attack complex (CAC) [54–56]. The two EV inhibitors MKT-077, and DMA-induced apoptosis in all target cells while omeprazole had little apoptotic effect in the tumor lines. MKT-077 and DMA also increased both paclitaxel- and cisplatin-induced apoptosis in MCF-7 and MDA-MB-231 cells. We obtained the same outcome in these studies and therefore examined the effect of the SMRwt peptides on complement mediated cytotoxicity in the three cell types. Treatment with the SMRwt peptides fostered reestablishment of complement-induced cell toxicity, and as discussed earlier, fostered antagonizing EV release and modifying EV protein content. This is consistent with preventing release of EV’s containing C9, thus leading to reestablishment of complement-mediated cytotoxicity. The SMR mutant peptides had no similar effect. Both SMRwt peptides were equally efficient in MCF-7 cells, but the PEG-SMRwt-Clu peptide was much more efficient in fostering this process in the MDA-MB-231 cells. Further, this effect on the complement system was consistent with observed SMRwt peptide effects on EV release and on EV content.

We also observed that treatment with the SMRwt peptides reduced expression of vimentin. High vimentin expression correlates with increased tumor growth, invasion, and poor prognosis [57]. Thus, reduced vimentin expression could be a factor in decreased tumor growth.

Finally, we found that knockdown of mortalin expression via mortalin-specific siRNA reduced mortalin protein levels, and that the resultant reduction of EV secretion reversed the block on complement-mediated cytotoxicity in all three tumor cell lines. Notably, knockdown of mortalin expression in K562 cells was not complete and as a result the complement-mediated killing was not as effective as it was in the other cell types.

Thus, the SMR peptides, through their antagonism of mortalin, block EV release and, in the tumor cell lines we examined, allow drug resistant tumor cells to reestablish the complement attack complex and complement mediated cytotoxicity of the target tumor cells, while having no similar effect on normal growing cells.

Mortalin is known to have tumor promoting and anti-apoptotic effects. Our results have shown that antagonism of mortalin in human K562, MCF-7 and MDA-MB-231 cells by SMRwt peptides allowed the tumor cells to reestablish complement attack, reduced tumor cell growth, and blocked EV release. These results suggest that SMRwt peptide antagonists of mortalin provide a possible avenue for anti-tumor therapy as well as being a potentially useful adjuvant in amplifying the therapeutic potential of tumor-targeted antibodies.

### Statistical analysis

A two-sample t-test assuming equal variances was used to compare the differences between controls and treated samples in each group. Data are expressed as the mean ± standard deviation (S.D.). A value of p ≤ 0.05 was considered statistically significant.

## MATERIALS AND METHODS

### Cell Lines, sera, chemicals and antibodies

MDA-MB-231, MCF-7, MCF-10A and K-562 cells were purchased from American Type Culture Collection (ATCC, Manassas, VA), cells were cultured in RPMI 1640 (Thermo Fisher Scientific, Rockford, IL) supplemented with 10% heat inactivated FBS purchased from MedSupply Partners (Atlanta, GA), 1% glutamine, 100 mg/ml penicillin, and 100 mg/ml streptomycin (Life Technologies, Carlsbad, CA) at 37° C and 5% CO_2_. Normal human serum (NHS) was purchased from MedSupply Partners (Atlanta, GA) as the source for the complement proteins. Heat-inactivated normal human serum (NHS) was prepared by heating serum at 56° C for 45 min. The rabbit polyclonal anti-mortalin antibody (anti-Grp75, catalog number: ab53098) and rabbit polyclonal to vimentin antibody (catalog number:ab45939) were purchased from Abcam, Inc., (Cambridge, MA), goat polyclonal anti-Alix antibody (catalog number: sc-49267) and donkey anti-goat IgG-HRP (catalog number: sc-2020) were purchased from Santa Cruz Biotechnology, INC (Dallas, TX), mouse monoclonal anti-a-Tubulin antibody (catalog number: T-5168) was purchased from Sigma-Aldrich, Inc., (Louis, MO). Peroxidase-conjugated goat anti-mouse IgG (catalog number: 31430), peroxidase conjugated rabbit anti-goat IgG (catalog number: 31460) were purchased from Thermo Fisher Scientific, Inc., (Rockford, IL). C9 antibody (catalog number: sc-9148) was purchased from Santa Cruz Biotechnology, Inc. (Dallas, TX).

### Peptides

The PEG-SMRwt-Clu, PEG-SMRmut-Clu, SMRwt-CPP and SMRmut-CPP peptides were custom made by InnoPep Inc. (San Diego, CA) (Figure 8).

### Mortalin HSPA9 DNA constructs

The BLOCK-iT Pol II miR RNAi expression vector kit from Life Technologies Corporation (Carlsbad, CA) and the mortalin (HSPA9) primers Hmi 408224 to Hmi 408227 were used to generate an expression vector with spectinomycin resistance (miR-mortalin) that expresses mortalin microRNA (miRNA; miR). The kit also contained a corresponding pcDNA6.2-GW/miR-neg control plasmid (miR-neg) predicted not to target any known vertebrate gene, which was used as a negative control.

### Cell proliferation and cytotoxicity assay

Human breast cancer cell lines MCF-7, MDAMB-231, and leukemia K562 cells, and non-tumorigenic MCF-10A cells are seeded into 96-well plates (5000 cells/well) and treated for 24 hours with various concentrations of SMR peptides including 0 - 1120 nM SMRwt-CPP and SMRmut-CPP. Cell proliferation is determined using the MTT {3-(4,5-dimet hylthiazol-2-yl)-2,5-diphenyltetrazolium bromide} dye assay by SpectraMax M5 Fluorescence Plate Reader (Molecular Devices. Sunnyvale, CA) as previous description^8^. Briefly, after cells were treated with SMR peptides for 24 hours, removed and discarded all supernatant, added 1x PBS to all wells for washing cells. 10 μL of yellow solution of MTT (stock conc. 5mg/mL) was added to 100 μL of phenol red-free medium in each well. The plates were incubated at 37° C for 4 hours for the reduction of MTT. Removed 85 μL form each well, added 50 μL of DMSO to each well and incubated at 37° C for 10 minutes and the absorbance was measured at 540 nm using a micro plate reader. Statistical significance of results is determined from three independent experiments including triplet or quadruplet sets in each experiment.

### Extracellular vesicle isolation and purification

Extracellular vesicles were isolated from breast cancer cells and leukemia cells by the miRCURY™ Exosome Isolation Kit (EXIQON, Woburn, MA), and instructions from the manufacturer to isolate EVs were followed. As a control, we used untreated tumor cells. Briefly, treated and untreated 10 mL of cell supernatants were centrifuged at 3000 × g for 15 minutes and then were centrifuged at 10,000 × g for 30 minutes and then using 0.22 μm filter to remove cell debris. The 10 mL of supernatants were transferred to a clear tube and added to 4 mL of precipitation buffer, mixed well and then incubated at 4° C for overnight. After incubation, the samples were centrifuged at 3200 × g for 30 minutes and the pellets were centrifuged at 3200 × g for 5 minutes. The pellet was washed by 1x PBS and was Ultra centrifuged at 100,000 xg for 2 hours. Finally, the EV pellets were re-suspended with PBS and stored at 4° C or −80° C until used for analysis.

### Extracellular vesicle characterization by acetylcholinesterase (AchE) assay

Purified EVs were quantitated by measurement of AchE as previously described, using a colorimetic assay with dithibionitrobenzoic acid (DTNB) as indicator [8]. After incubation with the acetylthiocholine iodide substrate, AchE activity was measured at 450 nm using a SpectroMax M5 fluorimeter.

### Protein analysis by Western blotting

Protein concentration was determined by measuring absorbance at 280 nm (Nanodrop 2000). Cultured cells were treated with PEG-SMR-Clu, SMR-CPP peptides, MKT-077, omeprazole and DMA at 37° C for 48 hr. Western blot analysis using anti-mortalin (Grp-75) antibody, anti-vimentin antibody and anti-Alix antibody expression of these proteins in EVs. For collection of proteins secreted from cells undergoing a complement attack, cells were treated with PEG-SMR-Clu and SMR-CPP peptides for 1 hour, after which was added anti-CXCR4 antibody in NHS or Hi-NHS for 1 hour at 37° C to stimulate the complement response. Then they were washed with PBS and were suspended in EV-free medium and were incubated at 37° C. After 1 hour, the cells were removed by centrifugation at 2000 × g for 10 min. Cell lysates and purified EVs were prepared by incubating 5–10 min at 95° C in sample buffer. The lysates were subjected to SDS-PAGE under reducing conditions (150 mM DTT) in a 4–20% criterion TGX precast gels and then transferred onto a nitrocellulose membrane (Bio-Rad, Richmond, CA). The membrane was blocked with 5% skim milk (MedSupply, Atlanta, GA) in TBS containing 0.05% Tween 20 (TBST) for 1 h at room temperature. The membrane was treated with anti-mortalin (Grp-75) mAb or anti-Tubulin mAb for cell lysates or anti- Alix and anti-vimentin or anti-mortalin for EVs and then with peroxidase-conjugated goat anti-mouse IgG, respectively. Bands were developed with an ECL chemiluminescent substrate (Santa Cruz Biotechnology, Santa Cruz, CA) and exposed to an ImageQuant LAS 4000 imaging system (GE Healthcare, Piscataway, NJ 08854). For determine expression of mortalin and C9 in MDA-MB-231, MCF-7 breast cancer cells and K562 cells, we treated cells with 600 nM PEG-SMRwt-Clu peptide or 300 nM SMRwt-CPP peptide alone in MCF-7 cells and 280 nM PEG-SMRwt-Clu peptide or 540 nM SMRwt-CPP peptide alone in MDA-MB-231 cells for 60 min at 37° C, followed by treatment of 1 μg/mL anti-CXCR4 antibody and 50 μL NHS or HIS for 60 min at 37° C, washing with PBS and the addition of fresh Exo-free medium incubated at 37° C for 60 min. Supernatants were collected and EVs were isolated. Released EVs were isolated and analyzed by SDS-PAGE and western blotting with anti-Alix, anti-Mortalin and anti-C9 antibodies.

### Apoptosis assay by flow cytometry

Assessment of apoptosis of breast cancer cells was seeded into 6-well plates at 4 × 10^5^ cells per well and treated with either paclitaxel or cisplatin as a positive control or various concentrations of SMR peptides or 337.5 nM MKT-077 on K562 cells, 300 μM DMA and 200 μM omeprazole alone or combined with 1.6 μM paclitaxel or 2 mg/mL of cisplatin for 48 hours. The cells were fixed with 4% paraformaldehyde in phosphate buffered saline (PBS) (pH 7.4). The terminal TUNEL assay was carried out following the manufacturer’s instructions (Nexcelom, Lawrence, MA) and measured by flow cytometry (GUAVA easyCyte HT, EMD Millipore Corporation, Temecula, CA).

### Complement-mediated cell cytotoxicity assay

Complement-mediated cell cytotoxicity assay was performed as described [53]. Briefly, MCF-7, MDA-MB-231 and K562 cells either were transfected with mortalin siRNA or without siRNA. Cells were incubated with 1 μg/ml of anti-CXCR4 antibody for 30 min at 4° C and then treated with PEG-SMRwt-Clu peptide, PEG-SMRmut-Clu peptide, SMRwt-CPP peptide, SMRmut-CPP peptide alone, treated with SMR peptide combined with complement from normal human serum (NHS) or heat inactivated normal human serum (NIS) for 60 min at 37° C. Percentage of cell lysis was determined with trypan blue by TC10 Automated Cell Counter (Bio-Rad, Richmond CA).

### Transient transfection with mortalin small interfering RNA (siRNA)

MDA-MB-231, MCF-7 breast cancer cells and K562 leukemia cells were plated in T-75 flasks with EV-free medium. After 24 hours in a 37° C incubator, the cells were collected. Cells were transfected with double-stranded siRNAs as described [8]. The small interfering RNA for HSPA9 and the SI-Neg control RNA were identical to that previously described [7, 8]. Cells treated with vector only (Mock) and with the siRNA-Neg construct (Neg) were used as controls. Following transfection, the cells were incubated in culture medium at 37° C for 72 hours before tested. Collected cells and EVs were isolated at 72 hours and measured by AchE assay and Western blotting.

### Nanoparticle tracking analysis (NTA) for extracellular vesicles

Analysis of the absolute size distribution in 300 μL of EVs was performed using NanoSight LM10 with NTA2.3 (NanoSight Ltd., Minton Park, UK). Particles are automatically tracked and sized based on Brownian motion and the diffusion coefficient. After isolation, the untreated and treated MDA-MB-231 and MCF-7 and K562 EVs were re-suspended in 0.5mL of PBS. Control medium and filtered PBS are used as controls in this technique. The NTA measurement conditions are described in Huang et.al [8]. Two recordings were performed for each sample.
